# Alteration of MicroRNAs Regulated by c-Myc in Burkitt Lymphoma

**DOI:** 10.1371/journal.pone.0012960

**Published:** 2010-09-24

**Authors:** Anna Onnis, Giulia De Falco, Giuseppina Antonicelli, Monica Onorati, Cristiana Bellan, Omar Sherman, Shaheen Sayed, Lorenzo Leoncini

**Affiliations:** 1 Department of Human Pathology and Oncology, University of Siena, Siena, Italy; 2 Aga Khan Hospital, Nairobi, Kenya; University of Barcelona, Spain

## Abstract

**Background:**

Burkitt lymphoma (BL) is an aggressive B-cell lymphoma, with a characteristic clinical presentation, morphology and immunophenotype. Over the past years, the typical translocation t(8;14) and its variants have been considered the molecular hallmark of this tumor. However, BL cases with no detectable *MYC* rearrangement have been identified. Intriguingly, these cases express *MYC* at levels comparable with cases carrying the translocation. In normal cells c-Myc expression is tightly regulated through a complex feedback loop mechanism. In cancer, *MYC* is often dysregulated, commonly due to genomic abnormalities. It has recently emerged that this phenomenon may rely on an alteration of post-transcriptional regulation mediated by microRNAs (miRNAs), whose functional alterations are associated with neoplastic transformation. It is also emerging that c-Myc modulates miRNA expression, revealing an intriguing crosstalk between c-Myc and miRNAs.

**Principal Findings:**

Here, we investigated the expression of miRNAs possibly regulated by c-Myc in BL cases positive or negative for the translocation. A common trend of miRNA expression, with the exception of hsa-miR-9*, was observed in all of the cases. Intriguingly, down-regulation of this miRNA seems to specifically identify a particular subset of BL cases, lacking *MYC* translocation. Here, we provided evidence that hsa-miR-9-1 gene is heavily methylated in those cases. Finally, we showed that hsa-miR-9* is able to modulate E2F1 and c-Myc expression.

**Conclusions:**

Particularly, this study identifies hsa-miR-9* as potentially relevant for malignant transformation in BL cases with no detectable *MYC* translocation. Deregulation of hsa-miR-9* may therefore be useful as a diagnostic tool, suggesting it as a promising novel candidate for tumor cell marker.

## Introduction

The c-Myc transcription factor is pathologically activated in many human malignancies [1]. A paradigm for c-Myc deregulation is offered by Burkitt Lymphoma (BL), where chromosomal translocations that join *MYC* with immunoglobulin (Ig) heavy- (Igh) or light-chain (Igκ, Igλ) *loci* are the crucial initiating oncogenic events [2]. High levels of c-MYC have been clearly shown to have a tumour-promoting effect [3]. Just a 2-fold difference in c-Myc expression can affect cell size in flies or cell number in mice [4]–[7].

However, there is increasing evidence that less than 10% of classical BL cases lack an identifiable *MYC* rearrangement [8]–[10]. Interestingly, no significant difference of *MYC* expression between *MYC* translocation-positive and negative cases has been found, independently of genomic alterations [10]. This may suggest that additional mechanisms, alternative to chromosomal translocations, which may result in *MYC* deregulation also exist. c-Myc expression is strictly regulated by a feedback loop autoregulatory mechanism involving the transcription factor E2F1, whose loss impairs *MYC*-mediated proliferation and lymphomagenesis [11]. c-Myc expression can be also regulated at the level of post-transcriptional control from a new class of non-coding RNA, miRNAs, able to inhibit mRNAs from being translated or causing them to be degraded [12].

MiRNAs play major roles in crucial processes as proliferation, differentiation and cell death [13]. It has become clear that alterations in the expression of miRNAs contribute to the pathogenesis of most, if not all, human malignancies [14]. MiRNA deregulation can be caused by various mechanisms, either genetic or epigenetic, including deletions, amplifications or mutations involving miRNA *loci*, epigenetic silencing or deregulation of transcription factors that target specific miRNAs [14].

In a previous study, we have analyzed a set of BL cases lacking *MYC* translocation, in which no other genomic aberrations, as increase of *MYC* copy number or aneuploidy were present, which showed high levels of *MYC* expression. We searched for alternative molecular alterations responsible for c-MYC deregulation in these cases and observed an altered expression of a specific miRNA, hsa-mir-34b, predicted to regulate *MYC* [10]. Being *MYC* a specific target of this miRNA, its deregulation may explain *MYC* altered expression in these cases [10].

However, recent literature reports that c-Myc itself is in turn able to activate the expression of several miRNAs [15]–[18] In particular, hsa-miR-17-5p and hsa-miR-20a are members of the miR-17-92 cluster, reported in literature as activated by c-Myc [15], [16]. In addition, the expression of both the functional strands 3′-end (miR-9) and 5′-end (miR9*) of the miRNA hsa-miR-9* has been recently described to be induced by c-Myc [17], [18].

In this study, we aimed at analyzing the expression of these specific miRNAs regulated by c-Myc in the previously described set of BL cases, based on the existence of a regulatory loop linking c-Myc and specific miRNAs. Our results show that a single miRNA, hsa-miR-9*, was found differentially expressed between BL cases carrying or not *MYC* translocation, being significantly down-regulated only in *MYC* translocation-negative cases. Intriguingly, we provide evidence that hsa-miR-9* is able to modulate E2F1 and c-Myc expression, suggesting down-regulation of hsa-miR-9* as a possible mechanism of c-Myc over-expression in BL cases negative for the translocation.

In summary, a better knowledge of miRNA alteration in such cases can potentially provide new markers to improve diagnosis and prognosis, as well as novel therapeutic approaches for BL treatment.

## Materials and Methods

### Ethics Statement

Ethics approval for this study was obtained from the Institutional Review Board at the University of Siena, University of Nairoby and at the CNIO. Informed written consent was obtained in all cases.

### Cases selection and Immunophenotype

For this study BL specimens collected at the Department of Pathology, Nairobi Hospital, Kenya and the Department of Human Pathology and Oncology, University of Siena, Italy, with available remaining materials from our prior study, have been used [10]. Since *MYC* translocation- negative BL cases may represent a challenging diagnosis to discriminate them from DLBCL and Intermediate DLBCL/BL, additional formalin-fixed and paraffin-embedded (FFPE) specimens of *MYC* translocation-negative Diffuse Large B cell Lymphoma (DLBCL), Intermediate DLBCL/BL and BLs have been retrieved at the Department of Human Pathology and Oncology, University of Siena, Italy, at the Molecular Pathology Programme, CNIO, Spain and at the Aga Khan University, Nairobi, Kenya. Attention was given to select cases with immunophenotype suggestive of BL (CD10+, BCL6+, BCL2- or weakly positive). Cases were reviewed by expert pathologists (BC, LL) and diagnoses were confirmed by morphology on histological slides stained with HE, Giemsa and by immunophenotyping, according to the Word Health Organization (WHO) classification which reports that *MYC* translocation-negative BL cases must be otherwise completely typical to make a diagnosis of BL [19].

Immunohistochemical studies were performed on representative paraffin sections from each case using microwave pre-treatment of slides for antigen retrieval. A large panel of antibodies recognizing formalin-resistant epitopes of the various antigens was applied, in conjunction with the alkaline phosphatase antialkaline phosphatase (APAAP) method, to visualize antibody binding [20]. Reactive lymph nodes were used as a control.

The presence of the Epstein-Barr virus (EBV) was assessed by *in situ* hybridization for EBERs using Epstein-Barr Virus (EBER) PNA/Fluorescein (DAKO, Denmark), a mixture of PNA probes complementary to the two nuclear EBER RNAs encoded by EBV, in conjunction with DAKO PNA ISH Detection Kit (DAKO, Denmark). Five micrometer-thick paraffin sections were deparaffinized, rehydrated and processed according to manufacturer's instructions. A control slide, prepared from a paraffin-embedded tissue block containing metastatic nasopharyngeal carcinoma in a lymph node, accompanied each hybridization run.

### Fluorescence *in situ* hybridization (FISH)

FISH analysis was performed following standard protocols used during EUROFISH and available at www.euro-fish.org. All reagents, instruments and split-signal probes were kindly provided by DakoCytomation (Glostrup, Denmark). Briefly, *MYC* rearrangements were sought using the *MYC* FISH DNA Probe-Split Signal using standard procedures. FISH was carried out as previously reported [10]. Dual color fluorescent *in situ* hybridization (FISH) for the *MYC* gene and the chromosome 8 centromere (CEN8), interpretation of results, classification of increased or not increased *MYC* gene copy number was carried out as previously described [10].

### Computational analysis

Messenger RNAs (mRNAs) predicted to be target of hsa-miR-9* were identified by computational analysis, using web-available resources (Mirnaviewer, PicTar, Tarbase [21] and miRBase [22]; mirnaviewer is available at http://cbio.mskcc.org/mirnaviewer; PicTar is a project of the Rajewsky lab at NYU's Center for Comparative Functional Genomics and the Max Delbruck Centrum, Berlin).

### MiRNA extraction

FFPE sections of primary tumors and reactive lymph nodes as a control were treated with Xylene to eliminate paraffin and then miRNAs were extracted using TRIZOL (Invitrogen, Milan, Italy). Germinal centre cells were isolated by laser capture microdissection and miRNAs were then extracted using TRIZOL. DNase I (Promega, Milan, Italy) treatment was performed in all samples.

### Analysis of miRNA expression

MiRNA expression was analyzed by qRT-PCR. Briefly, RNA samples were reverse transcribed using the Taqman MicroRNA reverse transcription kit (Applied Biosystems, Applera, Italy) and primers specific for each miRNA (hsa-miR-17-5p, hsa-miR-20a, hsa-miR-9, hsa-miR-9*), according to manufacturer's instructions. For each sample, 10 ng of total RNA were reverse transcribed. Real-time PCR was performed using Taqman probes specific for each miRNA and for RNU43, used as an endogenous control (Applied Biosystems, Applera, Italy). Amount and quality of RNA were evaluated measuring the OD at 260 nm, the 260/230 and the 260/280 ratios by Nanodrop (Celbio, Italy). Quality of RNA was also checked by BioAnalyzer (Agilent, CA).

### Gene expression analysis

Relative quantification of gene expression for E2F1 and *MYC* was also carried out by Real-time PCR using FluoCycle SYBR green (Euroclone, Celbio, Italy) according to manufacturer's instructions. HPRT was used as housekeeping gene. Primer sequences for *MYC* amplified a region of 129 bp: LEFT: AGCGACTCTGAGGAGGAAC; RIGHT: TGTGAGGAGGTTTGCTGTG. Primer sequences for E2F1 amplified a region of 136 bp: LEFT: GCCCTGAGGAGACCGTAG; RIGHT: ACAACAGCGGTTCTTGCTC. Primer sequences for HPRT amplified a region of 191 bp: LEFT: AGCCAGACTTTGTTGGATTTG; RIGHT: TTTACTGGCGATGTCAATAAG. Differences in gene expression were calculated using the ΔΔCt method [23].

### Cell lines and nucleofection

A human B lymphoblastoid cell line (LCL) was used to perform the *in vitro* experiments. Briefly, cells were cultured in RPMI supplemented with 10% FBS, 1% L-glutamine, penicillin/streptomycin, with 5% CO_2_, at 37°C. Transient transfections were performed by nucleofection, using an Amaxa apparatus, program A23 and solution V (Amaxa, Cologne, Germany). A transfection efficiency of 45% was obtained, as assessed by FACS analysis for a GFP reporter. Cells (2×10^6^) were transfected with 10 nM, 25 nM and 50 nM of hsa-mir-9* mimic (C-300115-04-0005, Dharmacon, Celbio, Italy), 50 nM miRNA inhibitor (I-300115-04-0005, Dharmacon, Celbio, Italy) or with 50 nM negative controls (NC I: IN-001000-01, NC: CN-001000-01, Dharmacon, Celbio, Italy). RNA was extracted 24 hours after nucleofection and hsa-miR-9* expression was checked by Real-Time RT-PCR, as previously described. Probes for qRT-PCR recognize both endogenus and transfected hsa-mir-9*.

### Western blotting

Cell pellets of LCLs, untransfected or transfected with either hsa-miR-9* 50 nM, its inhibitor or their respective negative controls, NC and I NC, were lysed in EBC buffer (50 mM Tris-HCl pH 8.0, NaCl 120 mM, 0,5% NP-40 and fresh protease inhibitors). Western blotting (WB) was performed with anti-E2F1 ([1∶200], KH95, sc-251, Santa Cruz), anti-c-Myc ([1∶200], 9E10, sc-40 Santa Cruz) and anti-actin ([1∶500], clone AC-40, #A-4700, Sigma Aldrich, Milan, Italy) using the ECL (Pierce, Rockford), following manufacturer's instructions.

### DNA extraction and BSP

FFPE section (10 µM) of 8 cases of endemic BL, negative for *MYC* translocation, and two reactive lymph nodes were deparaffinized with Xylene and DNA extraction was performed with NucleoSpin kit (Macherey-Nagel), according to manufacturer's instructions.

Amount and quality of DNA were evaluated measuring the OD at 260 nm, the 260/230 and the 260/280 ratios by Nanodrop (Celbio, Italy). DNA quality control PCR was also performed as previously described [24]. 1 µg of DNA of 7 cases was modified by bisulfite (Chemicon kit) for 15 hours as previously reported [25]. Approximately 100 ng of converted DNA were amplified using methylation-insensitive primers (no potential methylation site in their recognition sequence) for bisulfite sequencing as reported [26].

PCR products were separated on a 2% agarose gel to confirm size, quantity and purity of each product, and then purified using NucleoSpin Kit (Macherey-Nagel), according to manufacturer's instructions. 2 µl aliquot of purified PCR products were cycle sequenced using Big Dye Terminator Mix (Applied Biosystem, Foster City, CA) in a total volume of 20 µl. Reaction products were purified with ethanol/MgCl_2_ and finally 5 µl of product were resuspended in 15 µl of ultra pure water before being loaded on ABI capillary sequencer. Sequence reads were base-called using Chromas program. To explore *in silico* bisulfite modified DNA (either or not methylated at its CpG dinucleotides) methBLAST was used (http://medgen.ugent.be/methBLAST/), a sequence similarity search program.

## Results

### Clinical, morphological features and immunophenotype

BL, DLBCL and Intermediate BL/DLBCL cases were diagnosed based on morphology, immunophenotype and genetic alterations, in accordance to the WHO classification [19]. The clinical and pathological data of these cases are summarized in the Table 1.

**Table 1:** Clinical and pathological data of the different categories of analysed lymphoma.

	*MYC+ BL*	*MYC- BL*	*DLBCL GC-like*	*Intermediate DLBCL/BL*
**Age range, y (median)**	2–38 (14)	6–77 (17)	20–90 (49)	30–75 (31)
**Sex F/M**	3/7	3/6	6/4	2/4
**Site (nodal/extranodal)**	7/3	3/6	5/5	2/4
**EBV+/total**	5/10	1/9	0/10	0/6
**HIV+/total**	0/10	0/9	0/10	2/6
***MYC*** ** translocation**	+	−	−	−
Total	10	9	10	6

### c-Myc-induced miRNAs: hsa-miR-17-5p, hsa-miR-20a, hsa-miR-9 and hsa-miR-9*

The expression of hsa-miR-17-5p, hsa-miR-20a, hsa-miR-9 and hsa-miR-9*, reported to be regulated by c-Myc, was checked in all of BL cases. As expected, a strong up-regulation of hsa-miR-17-5p and hsa-miR-20a was found in all of the cases, independently of *MYC* translocation, which is in line with high expression of *MYC* in these cases (data not shown). Hsa-miR-9 showed an heterogeneous expression among all of BL cases, with no significant difference between *MYC* translocation-positive and negative BL cases (data not shown).

**Figure 1:**
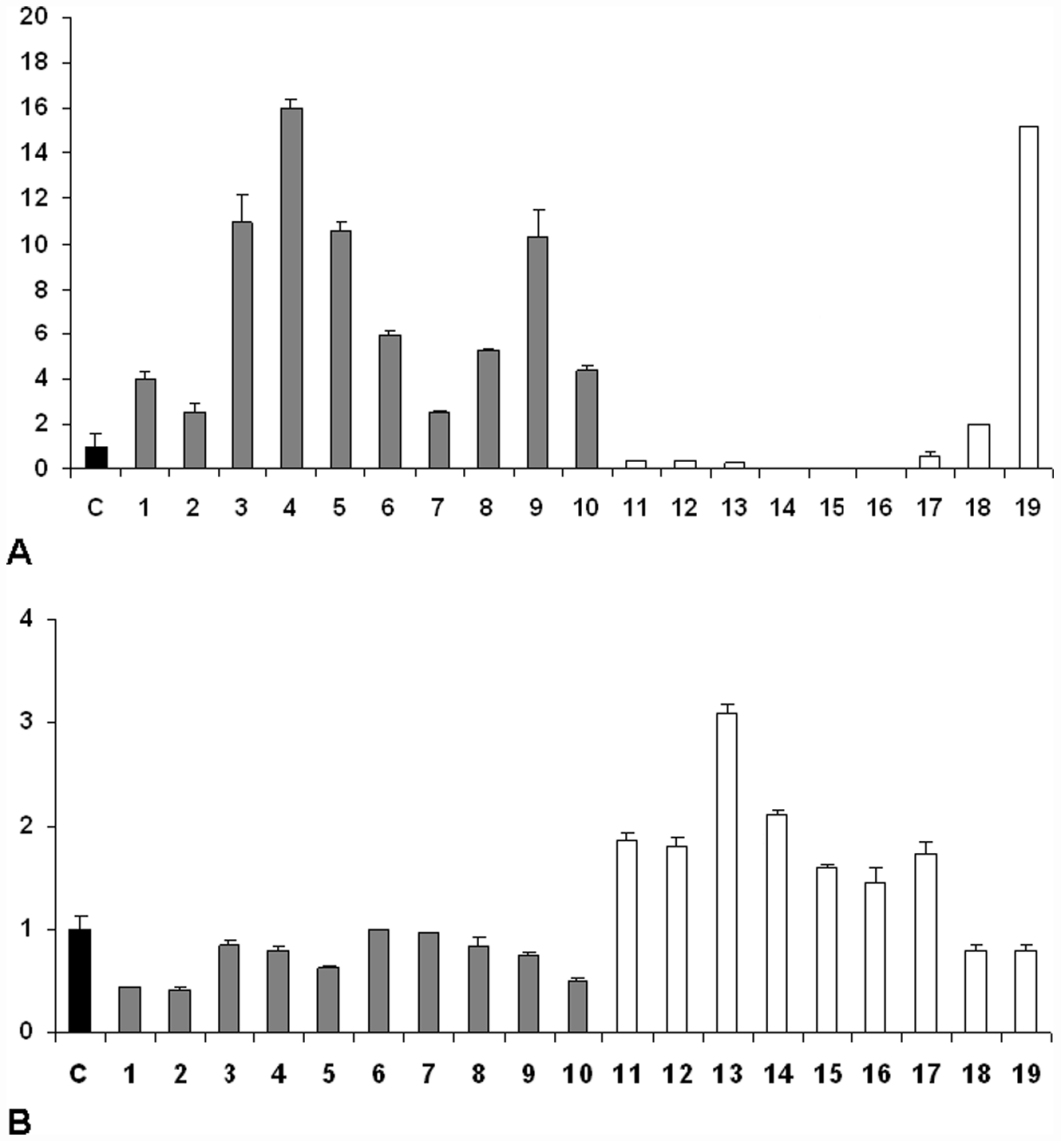
hsa-miR-9* and E2F1 expression in primary *MYC* translocation-positive and negative BL cases. (A) Expression of hsa-miR-9* in primary ***MYC*** **translocation-positive and negative BL cases.** Reactive lymph nodes and germinal centre cells (C); BL cases positive for *MYC* translocation [1]–[10]; BL cases negative for *MYC* translocation [11]–[19]; Relative expression of hsa-miR-9* was evaluated by qRT-PCR. BL cases positive for *MYC* translocation showed hsa-miR-9* up-regulated, whereas it was down-regulated in *MYC* translocation-negative ones, except for one case. **(B) Expression of E2F1 in primary** ***MYC*** **translocation-positive and negative BL cases.** Reactive lymph nodes and germinal centre cells (C); BL cases positive for *MYC* translocation [1]–[10]; BL cases negative for *MYC* translocation [11]–[19]; Relative expression by qRT-PCR for E2F1 in primary cases showed an up-regulation of E2F1 mRNA expression only in BL cases negative for *MYC* translocation. In all of the other cases, E2F1 mRNA levels were lower or comparable to control. Differences in gene expression were calculated using the ΔΔCt method. Results are representative of three different experiments. Error bars represent standard deviation between triplicates.

Interestingly, the expression of hsa-miR-9* was significantly different between *MYC* translocation-positive and negative cases, compared to normal controls (reactive lymph nodes and GC cells), being over-expressed in cases carrying *MYC* translocation (10/10) and strongly down-regulated in the others (8/9) (Figure 1A).

Of note, one case negative for the translocation expressed hsa-miR-9* at high levels. It is worth noting that among the cases lacking *MYC* translocation, this was the only case positive for EBV, and occurred in an elderly patient (Figure 1A).

**Figure 2:**
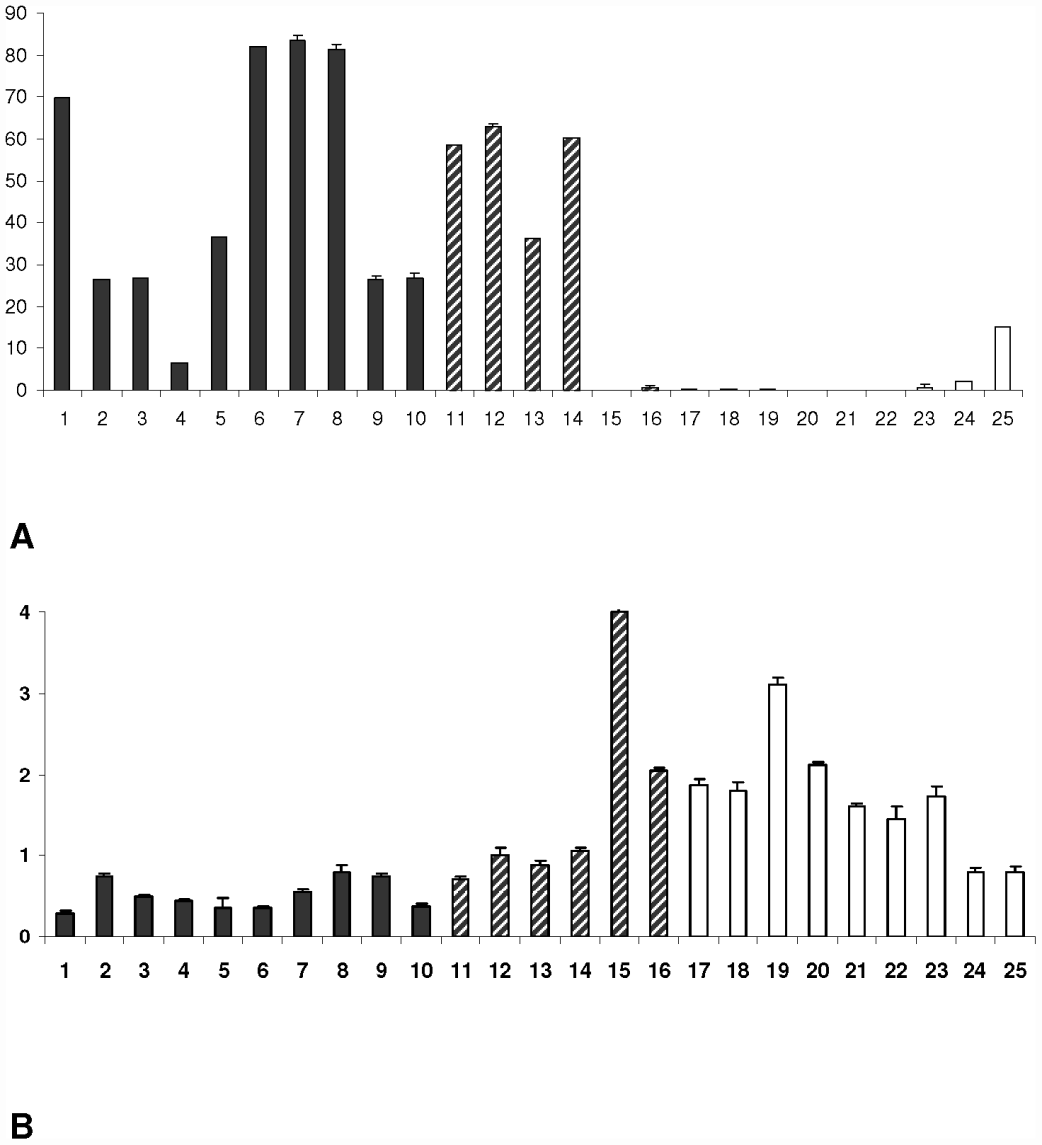
Expression of hsa-miR-9* and E2F1 in primary DLBCL and DLBCL/BL Intermediate cases in comparison with *MYC* translocation-negative cases. (A) Expression of hsa-miR-9* in primary DLBCL and DLBCL/BL Intermediate cases in comparison with ***MYC*** **translocation-negative cases.** DLBCL cases [1]–[10]; DLBCL/BL Intermediate cases negative for *MYC* translocation [11]–[16]; BL cases negative for *MYC* translocation [17]–[25]. Relative expression of hsa-miR-9* was evaluated by qRT-PCR. All of DLBCL cases showed hsa-miR-9* over-expression, in comparison with *MYC* translocation-negative BL cases. Intermediate DLBCL/BL cases showed an heterogeneous expression of hsa-miR-9*.**(B) Expression of E2F1 in primary DLBCL and DLBCL/BL Intermediate cases in comparison with** ***MYC*** **translocation-BL negative cases.** DLBCL cases [1]–[10]; DLBCL/BL Intermediate cases negative for *MYC* translocation [11]–[16]; BL cases negative for *MYC* translocation [17]–[25]. Relative expression of E2F1 was evaluated by qRT-PCR. E2F1 resulted less expressed in DLBCL cases, in comparison with *MYC* translocation-negative BL cases. Intermediate DLBCL/BL cases showed an heterogeneous expression of E2F1. Differences in gene expression were calculated using the ΔΔCt method. Results are representative of three different experiments. Error bars represent standard deviation between triplicates.

The absence of *MYC* translocation in BL cases may represent a challenge in the diagnosis of BL, to discriminate them from DLBCL and cases with intermediate features between DLBCL and BL cases (DLBCL/BL). To further confirm a role of hsa-miR-9* as a possible molecular marker for *MYC* translocation-negative BL, we tested its expression in DLBCL GC-like and Intermediate DLBCL/BL *MYC* translocation-negative cases. Hsa-miR-9* resulted over-expressed in all of DLBCL cases (10/10). As expected, Intermediate DLBCL/BL cases showed an heterogeneous expression of hsa-miR-9*. In particular, hsa-miR-9* was over-expressed in 4/6 cases, whereas 2/6 showed low expression, similarly to BL cases lacking *MYC* translocation (Figure 2A).

### E2F1 expression in primary tumors

We then investigated the molecular mechanism underlying such a difference in hsa-miR-9* expression. Using valid on-line database, we searched for mRNAs predicted to be hsa-miR-9* targets. Among those predicted, we found E2F1 as a possible intriguing candidate, because it is known to controls c-Myc expression [27], [28]. First, we tested its expression in *MYC* translocation-negative BL cases in comparison to BL carrying *MYC* translocation and normal controls (reactive lymph nodes and GC cells). E2F1 was up-regulated only in BL cases lacking *MYC* translocation, in respect with both BL cases carrying *MYC* translocation and normal controls (Figure 1B). To further confirm these results, we tested its expression also in DLBCL and Intermediate DLBCL/BL cases (Figure 2B), where an inverse correlation between hsa-miR-9* and E2F1 was also found. Table 2 summarizes the level of the expression of hsa-miR-9* and E2F1 with the presence or not of *MYC* translocation in the different categories of analysed lymphomas.

**Table 2:** Summary of miRNA analysis in the different categories of analysed lymphoma.

	*MYC+ BL*	*MYC- BL*	*DLBCL GC-like*	*Intermediate DLBCL/BL*
***MYC*** ** translocation**	+	−	−	−
Hsa-miR-9* expression level (median fold changes)	High (5.57)	Low (0.352)	High (31.6)	High/Low (47.3/0.25)
**E2F1 expression level (median fold changes)**	Medium (0.78)	High (1.74)	Medium (0.5)	Medium/High (0.78/3.02)
Total	10	9	10	6

The median fold changes has been used as an indicator of the expression level in each category of lymphomas. To group the median fold changes, cut-offs have been defined as low (median fold changes <0.5), medium (median fold changes between 0.5 and 1.5), and high (median fold changes >1.5) expression level.

E2F1 over-expression observed in *MYC* translocation-negative BL cases could be responsible for c-Myc over-expression, and to sustain it at high levels in the absence of translocation. This event may be relevant for malignant transformation, because of the reciprocal activation E2F1/c-Myc.

### Functional *in vitro* study on hsa-miR-9*

**Figure 3:**
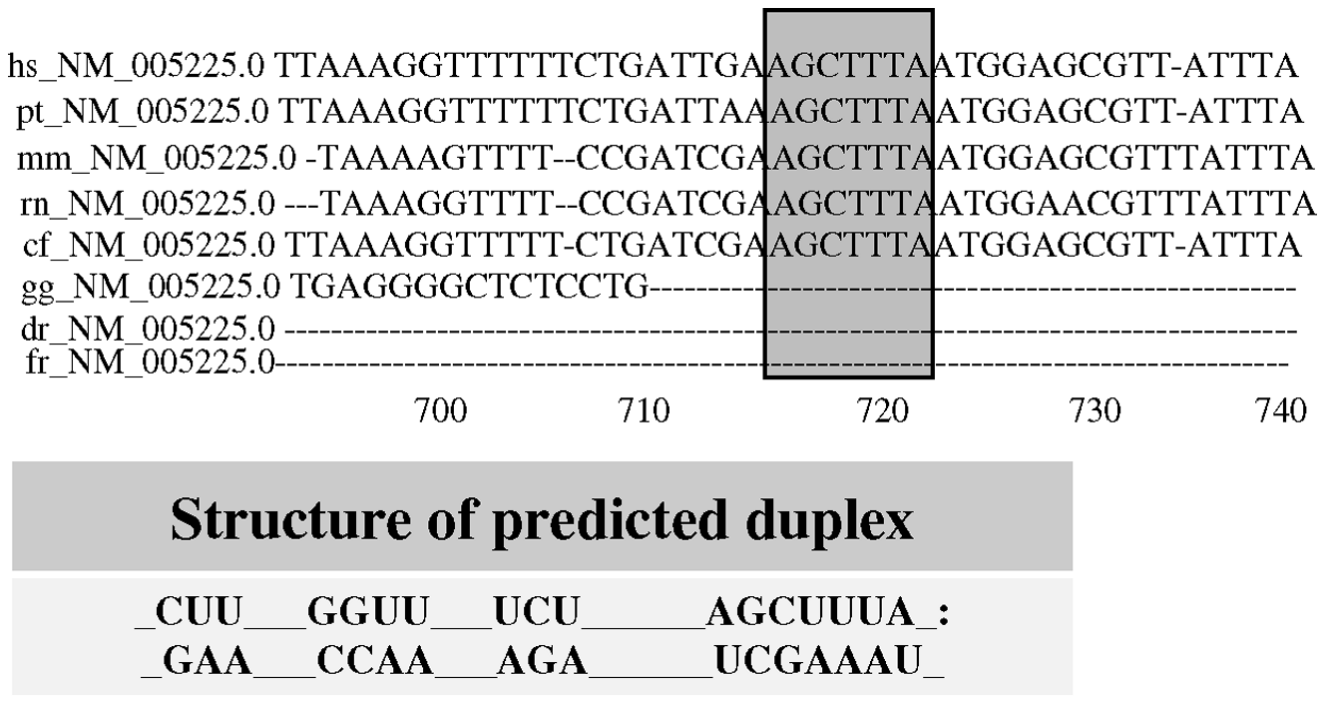
Computational analysis of E2F1 binding to hsa-miR-9*. Alignment of the predicted hsa-miR-9* target site in the E2F1 3′-UTR from several different species as predicted by PicTar. Shaded boxes indicate bases pairing with hsa-miR-9*. On the bottom of the figure the structure of predicted duplex in human specie has been reported.

**Figure 4:**
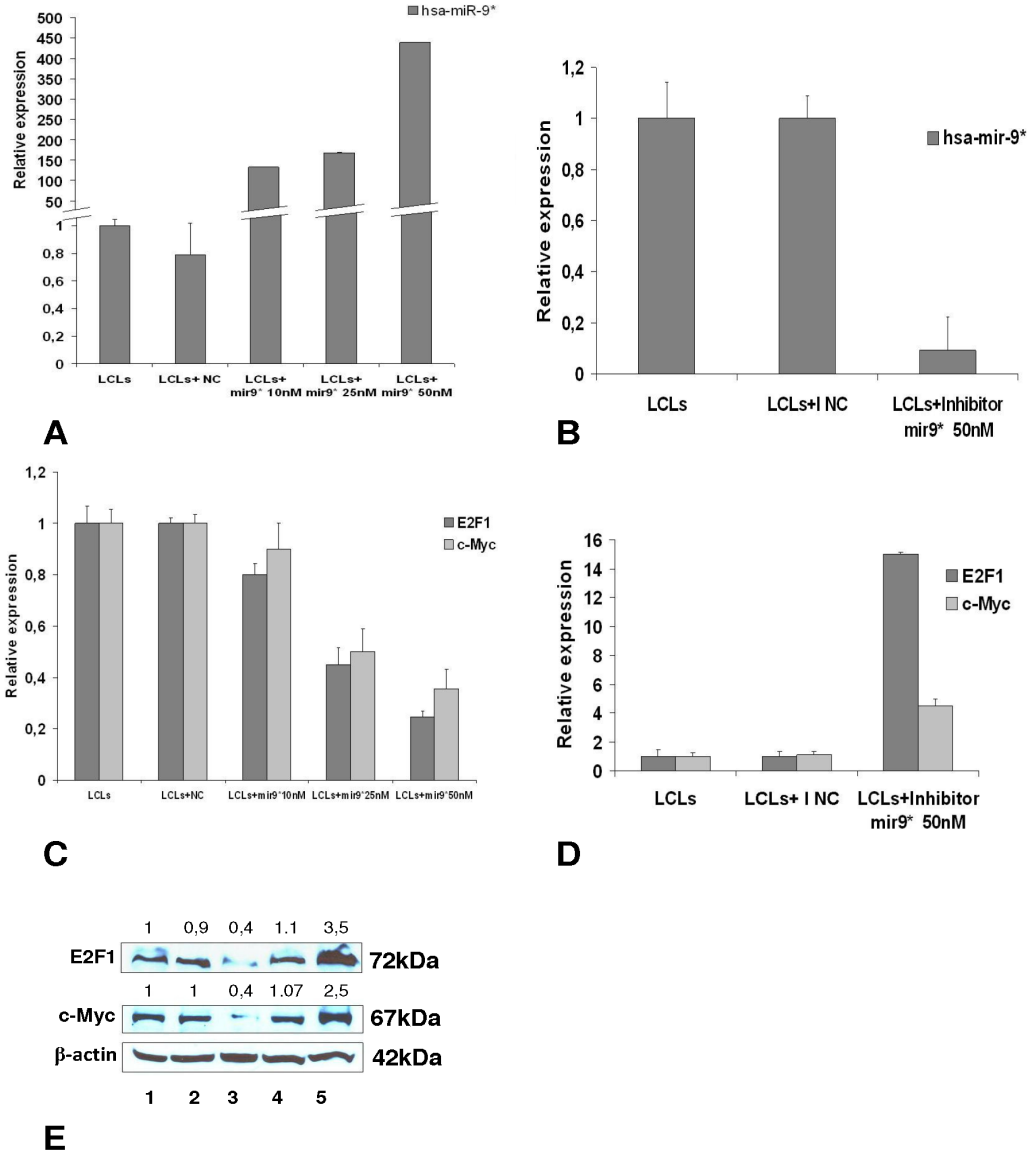
Functional *in vitro* study on hsa-miR-9*. (A) Relative expression by qRT-PCR of hsa-mir-9* in LCL transfected with increasing amounts of a synthetic hsa-mir-9*, compared to non-treated LCL and LCL transfected with a negative control (NC). The level of hsa-mir-9* increased in a dose-dependent manner. (B) Relative expression by qRT-PCR of hsa-mir-9* in LCL transfected with an hsa-miR-9* inhibitor, at the concentration of 50 nM, compared to non-treated LCL and LCL trasfected with a negative control (I NC). The level of hsa-mir-9* decreased significantly upon transfection of the inhibitor. **(C) Relative quantification of E2F1 and** ***MYC*** transcripts after transfection of increasing amounts of a synthetic hsa-mir-9*. A dose-dependent decrease of both genes is detected in the presence of synthetic hsa-mir-9*. **(D) Relative quantification of E2F1 and** ***MYC*** transcripts after transfection of a hsa-mir-9* inhibitor. Transfection of the hsa-mir-9* inhibitor is able to increase E2F1 and *MYC* expression, in comparison with the expression level of both proteins in non-treated LCL or cells transfected with a negative control (I NC). (E) E2F1 and c-Myc protein levels in LCL transfected with higher concentration of synthetic hsa-mir-9*, hsa-mir-9* inhibitor and their negative controls, evaluated by western blotting. Lane 1: non-treated LCL; lane 2: LCL transfected with NC, lane 3: LCL transfected with 50 nM synthetic hsa-mir-9*, lane 4: LCL transfected with I NC; lane 5: LCL transfected with 50 nM hsa-mir-9* inhibitor. Figure is representative of three different experiments. Numbers on top indicate densitometric analysis.

To confirm the bioinformatic predictions which indicate E2F1 as a possible target of hsa-miR-9* (Figure 3), we transfected a synthetic hsa-mir-9* (Figure 4A) or its inhibitor (Figure 4B) in a human B lymphoblastoid cell line, and then measured E2F1 expression both at the mRNA and protein levels by qRT-PCR and western blotting, respectively. We found a significant dose-dependent decrease in E2F1 mRNA in cells transfected with hsa-mir-9* (Figure 4C). Conversely, when a hsa-mir-9* inhibitor was transfected, an increase in E2F1 mRNA was observed (Figure 4D).

As c-Myc expression can be induced by E2F1, we also checked *MYC* mRNA expression. Accordingly, *MYC* mRNA levels resulted down-regulated after transfection of a synthetic hsa-miR-9* (Figure 4C). On the other hand, we observed an increase of *MYC* mRNA expression after silencing of hsa-miR-9* (Figure 4D).These findings were confirmed at the protein level, by western blotting (Figure 4E).

### Methylation analysis of hsa-miR-9-1 gene in primary tumors

**Figure 5:**
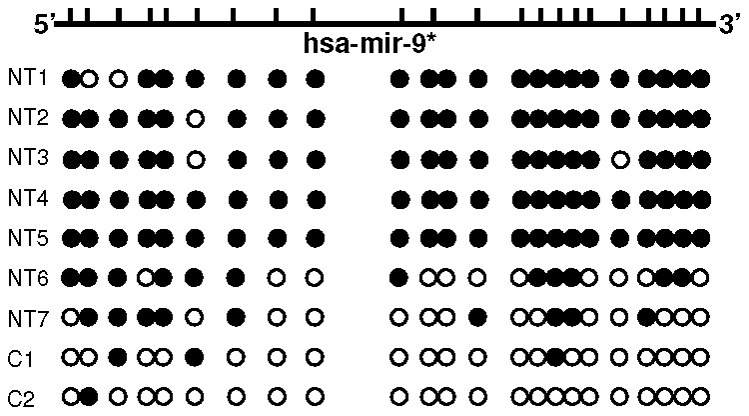
Methylation analysis of hsa-mir-9-1 gene in primary tumors. Methylation density of hsa-mir-9-1 gene in endemic BL cases negative for *MYC* translocation (NT, Negative for translocation) and reactive lymph nodes (C1, C2) by bisulfite sequencing; Solid circles (•) and empty circles (○) indicate that CpG site is methylated or unmethylated, respectively. The BL cases negative for *MYC* translocation showed aberrant (cases 1–5) and partial (cases 6 and 7) methylation, in respect with the normal controls (C1 and C2). Cases NT1 and NT2 showed 21/23 CpG methylated, case NT3 22/23 CpGs methylated, cases NT3 and NT4 showed all of CpGs methylated (23/23), case NT5 showed 12/23 CpG methylated and case NT6 showed 9/23 CpG methylated. On the other hand, the normal controls (C1, C2) showed 2/23 and 1/23 CpGs methylated, respectively.

Hypermethylation is described as possible mechanism of miRNA silencing. Due to the presence of a CpG island in hsa-miR-9-1 gene (Ensemble Genome browser, ENSG00000207933), we performed methylation analysis by bisulfite sequencing to assess whether down-regulation of hsa-miR-9-1 in those cases could rely on an epigenetic inactivation. Figure 5 shows the results, indicating hypermethylation in BL cases negative for *MYC* translocation, in respect with normal controls.

## Discussion

The molecular feature of Burkitt lymphoma (BL) is the translocation that places *MYC* under the control of immunoglobulin gene regulatory elements. However, there is recent evidence that infrequent cases may lack an identifiable *MYC* translocation, the explanation for which is still uncertain, though suggesting the existence of pathogenetic mechanisms alternative to genetic alterations [8], [9].

Recently, it has been suggested that the over-expression of *MYC* in the absence of translocation might be due to miRNA deregulation [10]. It has been recently demonstrated that hsa-miR-34b is expressed at different instances between *MYC* translocation-negative and positive BL cases [10]. In particular, it is down-regulated only in *MYC* translocation-negative BL cases, giving evidence that its deregulation could influence c-Myc expression in the absence of genomic translocation. On the other hand, c-Myc itself is able to activate the expression of specific miRNAs [12]–[15], suggesting the existence of a feedback loop between c-Myc and specific miRNAs, able to reciprocally control their expression. It may then be speculated that c-Myc over-expression induces a specific miRNA pattern that, in turn, might be responsible for a differential gene expression, and for functional alterations of tumor cells.

Here, we found a strong up-regulation of hsa-miR-17-5p and hsa-miR-20a, which correlates with high levels of *MYC* expression in these tumors, and is in line with the c-Myc-mediated induction of the miR-17-92 cluster. An heterogeneous expression of hsa-miR-9 was observed among BL cases, whereas hsa-miR-9* was the only miRNA strongly down-regulated only in BL *MYC* translocation-negative cases, with the exception of one case. Interestingly, this case was the only EBV-positive among the *MYC* translocation-negative BL cases. It might be argued that the virus may be able to modulate hsa-miR-9* expression in the absence of translocation, being now clear the existence of a delicate and complex interplay between viruses and cellular miRNAs [29]–[32].

The *MYC* translocation-negative BL cases may represent a challenging diagnosis to discriminate them from DLBCL and from cases with intermediate features between DLBCL and BL cases (DLBCL/BL) [8], [19], [33], [34]. The latter is an heterogeneous category that is not considered a distinct disease entity, but it is useful in allowing the classification of cases not meeting criteria for classical BL or DLBCL. The diagnosis should not be made in cases of morphologically typical DLBCL that have a *MYC* rearrangement, or in otherwise typical BL in which a *MYC* rearrangement cannot be demonstrated. Improvements in appropriate therapeutic approaches should be established for this category which as extremely poor prognosis.

To further confirm that the hsa-miR-9* may be considered specific a molecular marker for BL cases lacking the typical translocation, we tested its expression also in DLBCL and Intermediate DLBCL/BL cases. We found that DLBCL cases showed a strong over-expression of hsa-miR-9*, whereas Intermediate DLBCL/BL cases an heterogeneous expression. In fact, among the latter hsa-miR-9* was over-expressed in 4/6 and down-regulated in 2/6, similar to BL cases lacking *MYC* translocation. These findings further suggest that Intermediate DLBCL/BL cases are an heterogeneous category and additional studies are necessary to demonstrate whether hsa-miR-9* may be used as a specific marker to differentiate BL from DLBCL, and to identify cases that may benefit from a more aggressive therapy.

To unravel the pathological role of hsa-miR-9* down-regulation on *MYC* translocation-negative BL cases, we looked at mRNAs predicted to be target of hsa-miR-9*. Among those predicted, we found particularly interesting E2F1, which is essential for the G1-S1 phase passage. Expression of E2F1 is known to be induced by c-Myc, and in turn controls c-Myc expression [27], [28].

Interestingly, an inverse correlation between hsa-miR-9* and E2F1 was found. In particular, E2F1 over-expression observed in BL cases lacking the typical translocation may represent itself an appealing possible mechanism able to determine c-Myc over-expression and sustain it at high levels in the absence of translocation, due to the existence of a feedback loop responsible for controlling the reciprocal expression of these transcription factors. On the other hand, hsa-miR-9* up-regulation observed both in *MYC* translocation-positive BL, DLBCL and in 4/6 Intermediate BL/DLBCL cases may be consistent with a regulatory loop between hsa-miR-9* and E2F1. To verify our hypothesis, based on bioinformatic predictions, functional *in vitro* studies were performed. E2F1 regulation by hsa-miR-9* was observed, either direct or indirect, as miR9* ectopic up-regulation reduces E2F1 levels. Conversely, miR9* silencing induces E2F1 expression. As a consequence, also the expression of *MYC* was affected, both at the mRNA and protein levels. These findings highlight that hsa-miR-9* inactivation may determine E2F1 up-regulation and consequent *MYC* over-expression in BL lacking *MYC* translocation.

MiRNA silencing in cancer can be due to several mechanism, as a consequence of genomic abnormalities or epigenetic regulation [35]. Hsa-miR-9* down-regulation in *MYC* translocation-negative cases is due to an epigenetic mechanism, as an aberrant methylation of hsa-miR-9-1 gene was observed in these cases. Accordingly, inactivation of hsa-miR-9-1 gene due to hypermethylation has been suggested as an additional mechanism for miRNA inactivation also in human breast cancer [26].

**Figure 6:**
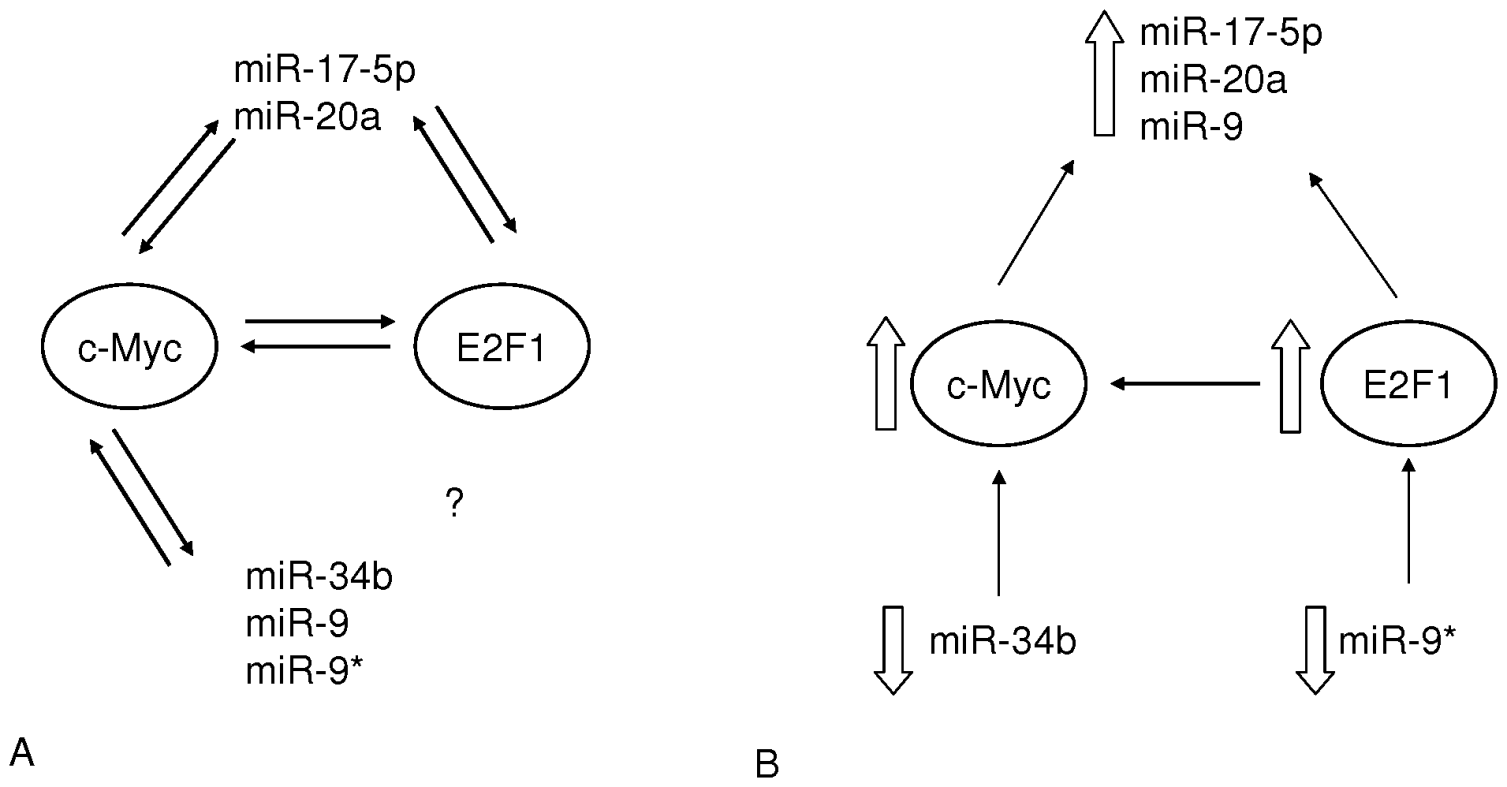
Models of regulatory interactions involving c-Myc, E2F1 and miRNAs. **(A) Regulatory network between c-Myc, E2F1 and miRNAs.** The interactions among c-Myc, E2F1 and miRNAs are shown. Bidirectional arrows indicate a transcriptional regulation. MiR-17-5p, miR-20a, miR-9, miR-9* and miR-34b are involved on c-Myc activation, on the other hand c-Myc itself regulates their expression. Further, miR-17–5p and miR-20a have been shown to inhibit E2F1 translation. E2F1 can induce miR-17-5p and miR-20a expression. **(B) Proposed model involving c-Myc, E2F1 and miRNAs in** ***MYC*** **translocation-negative BL.** MiR-34b and miR-9* down-regulation induces c-Myc expression directly or by E2F1 induction, respectively. c-Myc over-expression possibly determines up-regulation of miR-17-5p, miR-20a and miR-9.

Overall, we can conclude that several mechanisms may explain *MYC* over-expression. In particular, miRNA deregulation may have an important role in the pathogenesis of BL cases negative for *MYC* translocation, and may provide a new intriguing molecular mechanism other than typical translocation in affecting *MYC* expression (Figure 6).

Hsa-miR-9* down-regulation seems to be specific for BL cases lacking *MYC* translocation, and identifies hsa-miR-9* as a possible novel candidate for a more accurate clinical diagnosis, helping to design miRNA-based gene therapy, which could be the future tool for gene therapy.
